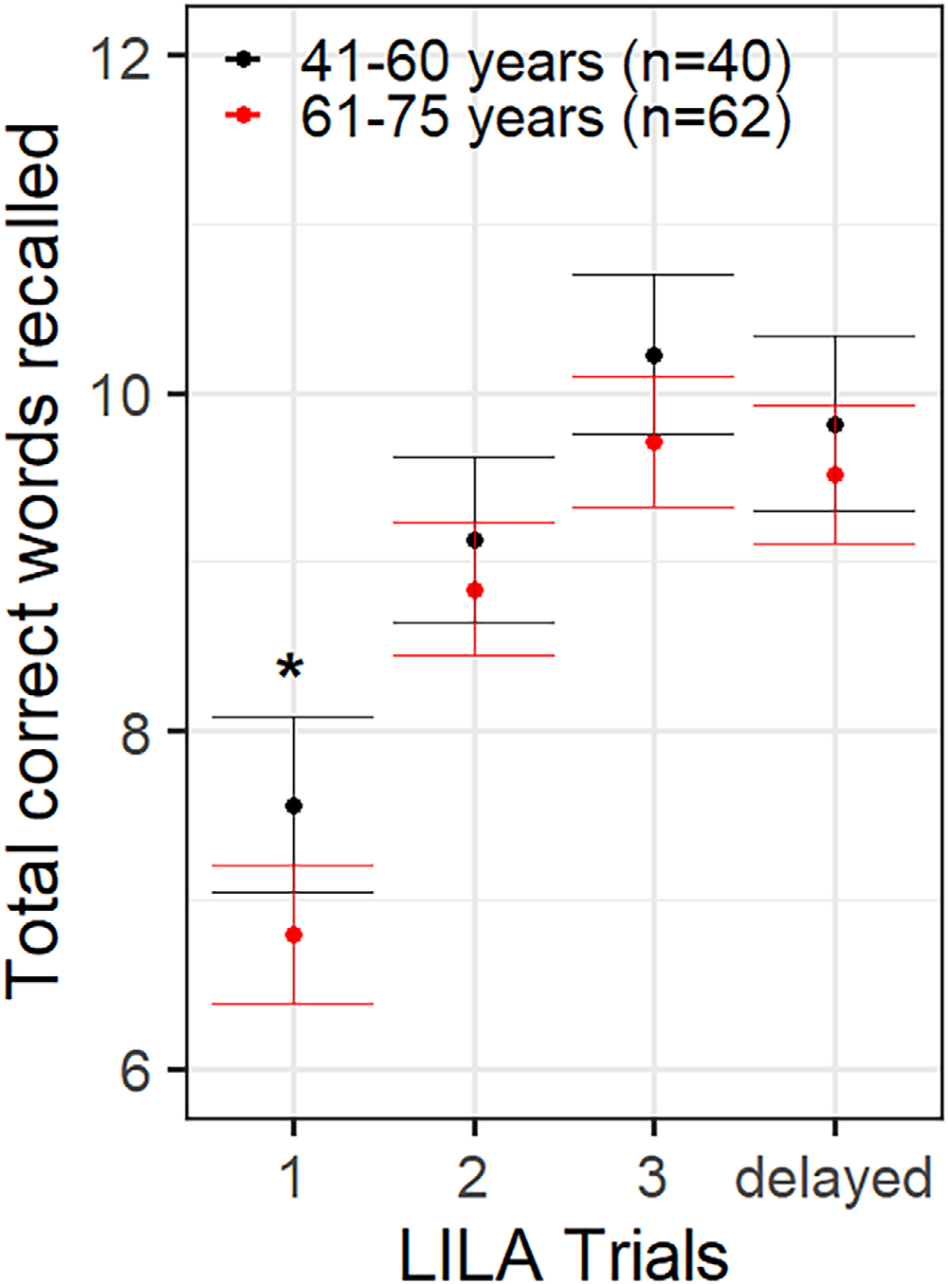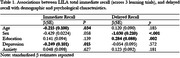# Acceptability and validity of a smartphone‐administered word list learning test, LILA, in cognitively unimpaired adults

**DOI:** 10.1002/alz.091888

**Published:** 2025-01-03

**Authors:** Yen Ying Lim, Hannah Cummins, Lisa Bransby, Gabriel Stellman, Amy Fredrickson, Chris J. Edgar, Paul Maruff

**Affiliations:** ^1^ Turner Institute for Brain and Mental Health, School of Psychological Sciences, Monash University, Melbourne, VIC Australia; ^2^ Turner Institute for Brain and Mental Health, Monash University, Melbourne, VIC Australia; ^3^ Cogstate Ltd, Chicago, IL USA; ^4^ Cogstate Ltd, Melbourne, VIC Australia; ^5^ Cogstate Ltd., London United Kingdom; ^6^ Cogstate Ltd., Melbourne, VIC Australia

## Abstract

**Background:**

The International Shopping List Test (ISLT) is a rater‐administered verbal list learning test, sensitive to memory dysfunction in early Alzheimer’s disease (AD). This study examined the acceptability of a self‐administered ISLT (called LILA) in cognitively unimpaired (CU) middle‐aged and older adults. The validity of LILA was determined by the nature of ISLT learning curves and from examination of the effects of age.

**Method:**

CU adults (n = 113) completed LILA on their own smartphone device in a remote, unsupervised setting (n = 113). The LILA app administered a novel ISLT, where a list of 12 shopping list items was read aurally through the speaker to the participant. Participants were required to recall, into the smartphone, as many words as they could remember. This immediate recall trial was repeated three times (T1‐3). After a 10‐minute delay, participants recalled as many of the 12 words as they could. LILA performance measures included, number of words recalled on each trial, and on the delayed recall trial.

**Result:**

10% of participants (n = 11) failed to provide a response on at least one learning trial (T1 = 3, T2 = 2, T3 = 4, DR = 2), with this data excluded from subsequent analyses. All participants reported feeling highly (74%) or moderately (26%) confident about using a smartphone, and reported using their phone daily. Improvement in immediate recall, known to occur for the ISLT was observed for LILA (Fig 1). Lower LILA immediate recall scores were associated with older age and higher depressive symptomatology (Table 1), but not sex, education or anxiety symptoms. Lower scores on delayed recall was associated with male sex and lower years of education (Table 1), but not age or mood symptoms.

**Conclusion:**

The very low rate of data loss indicates high acceptability of LILA in CU middle‐ and older‐aged adults. Validity of performance was indicated by the learning curves, which were qualitatively similar to those of the rater‐administered ISLT, and the effect of age on the immediate recall outcome. These data provide a strong foundation for future studies of the sensitivity of LILA to memory impairment in AD.